# Research on the Microscopic Aging Characteristics of Asphalt Binder Based on Atomic Force Microscopy

**DOI:** 10.3390/polym17081000

**Published:** 2025-04-08

**Authors:** Wenhui Li, Peiwen Hao, Guofeng Liu, Zhigang Li, Chen Le, Chun Wang, Wenduo Ma, Shaohui Li

**Affiliations:** 1 School of Highway, Chang’an University, Xi’an 710064, China; lwh111988@126.com (W.L.); 2019221162@chd.edu.cn (C.L.); wangchun0618@chd.edu.cn (C.W.); 2023221358@chd.edu.cn (W.M.); shl@chd.edu.cn (S.L.); 2 Yunnan Ningyong Expressway Co., Ltd., Lijiang 674300, China; ynnygsglgs@163.com; 3 School of Civil and Architectural Engineering, Xi’an University of Science and Technology, Xi’an 710064, China; lizhigang@xust.edu.cn

**Keywords:** road engineering, UV aging, AFM, microstructure, nonlinear optimization algorithm, aging performance prediction

## Abstract

To deeply analyze the difference between styrene–butadiene–styrene (SBS)-modified asphalt and base asphalt in ultraviolet (UV) aging performance, an atomic force microscope was used to carry out an accurate analysis of the bee structural characteristic parameters of two kinds of asphalt experiencing different UV aging time duration. With the advantages of the Levenberg–Marquardt (LM) algorithm and universal global optimization (UGO) algorithm, the relationship between the microscopic characteristic value and the performance index of asphalt under UV aging was constructed. The results show that the physical parameters of the bee structure of asphalt are closely related to the macroscopic properties of asphalt materials. Because the number of bee structures of SBS-modified asphalt is significantly more than that of base asphalt, and in the process of UV aging, the absolute height and area of the bee structure fluctuate less, and the adhesion force fluctuates with aging time. The decreasing trend makes SBS-modified asphalt significantly better than base asphalt in anti-UV aging performance. Under different UV aging time durations, the penetration and aging index (AI) of the two kinds of asphalt showed an excellent linear relationship with the microscopic indexes measured by atomic force microscope (AFM). Through the nonlinear optimization algorithm, the prediction equations of the morphology height and adhesion force of the two kinds of asphalt under different aging stages were successfully derived. The research results can provide theoretical support for the optimization and improvement of the UV aging performance of SBS-modified asphalt.

## 1. Introduction

In modern transportation infrastructure construction, asphalt has become the main material for road building. It has advantages such as good pavement performance, smoothness, noise reduction, and ease of construction [1,2]. As an important link connecting individual lives and work, the integrity of the structure and performance of asphalt pavements during their service life is a crucial factor in ensuring driving comfort [3].

However, asphalt pavements exposed to the natural environment inevitably suffer from erosion due to various environmental factors [4]. The aging of asphalt under environmental and climatic effects is the main internal cause of the decline in pavement performance [5]. UV radiation, an important aging factor, when acting on the pavement for a long time, can significantly reduce the deformation ability of asphalt and increase its brittleness. As a result, diseases such as cracks and spalling occur prematurely on asphalt pavements [6,7]. With the construction of China’s western region, the road network is gradually improving. But the unique strong UV environment in the plateau areas accelerates the aging of asphalt pavements [8,9], causing significant changes in the physical and chemical properties of asphalt materials [7,10,11]. This not only reduces the service level of roads but also increases road maintenance costs and traffic safety risks. Therefore, exploring the aging mechanism of asphalt is of great significance for facilitating the construction of asphalt pavements in the alpine and high altitude areas in the western region [12].

In order to improve the aging performance of asphalt binder, various measures were tested and compared, such as fiber addition [8], SBS modification [13], and Zinc Oxide (ZnO) modification, and their anti-aging performances were verified. However, due to the complexity of asphalt composition, the aging rate of asphalt varies greatly at different aging times. The aging rate of asphalt materials is the fastest in the initial stage of aging. The aging speed gradually slows down, as the UV aging time increases, and then increases again [14]. Affected by the asphalt aging process and aging rate, the pavement performance of asphalt mixtures also shows different degrees of decline patterns. The high temperature performance of asphalt mixtures is the most sensitive to changes in the UV aging environment, followed by fatigue performance, while the low temperature performance is less affected by UV aging [15]. The decay rate of water stability gradually decreases as the UV time increases until the water stability performance completely disappears [16].

Although existing macroscopic performance testing methods, such as evaluating the aging status of asphalt through indicators like penetration, viscosity, and rheological characteristics, have been widely used [17,18], microscopic chemical methods are widely applied in exploring the UV aging mechanism of asphalt from a microscopic perspective [2] and to explain the evolution law of the microscopic structure of asphalt during UV aging. For instance, fluorescence microscopy test, infrared spectroscopy test [19], gel permeation chromatography analysis [20,21], and Fourier infrared spectroscopy [22] were used to determine the microscopic structure of SBS-modified asphalt and the relative molecular mass and the four-component content of asphalt before and after its aging.

In addition, atomic force microscope (AFM) plays a vital role in the field of materials science and it has excellent nanoscale resolution and diverse detection modes to determine the aging degree of asphalt in its meso-structure surface, including the microscopic morphology, roughness, and mechanical properties of the material surface [23,24], among which bee structure is mostly often used [25]. Sizes of bee structures from tens of nanometers to hundreds of nanometers could be measured and based on this, four components of asphalt were determined further. The asphaltenes are white, the resins are yellow, the saturates are black, and the smooth areas are aromatics [24]. By analyzing the characteristic information of the bee structure before and after aging, the influence of each component on the aging degree of asphalt can be further judged [26]. In addition, basic performance was confirmed to have a good relationship with its microscopic morphology determined by AFM [9].

Influenced by the aging effect, the bonding performance of asphalt gradually decreases as the aging degree deepens [27]. The mode of UV aging affects the degree of asphalt aging. Wang et al. [28] designed indoor tests of indirect UV and continuous UV to compare the differences between the two UV action methods. By observing the morphological characteristics of the bee structure, they found that the morphology of each layer of asphalt aged by continuous UV light is similar to the bee structure of unaged asphalt. After intermittent UV light aging, the smoothness of the asphalt surface decreases. Although asphalt molecules do not age in an environment without UV light, the diffusion behavior of aged asphalt molecules does not stop, which deepens the degree of asphalt aging. Fang et al. [27] used the AFM method to explore the structural morphology of polyurethane-modified asphalt after short- and long-term aging. They found that adding polyurethane can effectively reduce the diffusion of aged asphalt molecules. The changes in its surface height and modulus are much smaller compared to those of base asphalt. This proves that polyurethane-modified asphalt has excellent anti-aging properties.

In recent years, with the growing power of machine learning and deep learning algorithms, which can accurately process and analyze data results, seeking the relationship between test conditions and the performance degradation of asphalt mixtures has gradually become a research hotspot. Moreover, predictive models are favored because they can reduce test costs and shorten test time [29,30]. For example, based on improved gray wolf optimization (IGWO), natural gradient boosting (NGBoost) [31], Newton–Raphson root-solving algorithm [32], and the LM method [33] were used to determine pavement friction coefficient, to evaluate its remaining life, and to predict pavement cracking.

In summary, analyzing the microscopic data obtained from AFM can effectively and accurately evaluate the aging process of asphalt materials. Therefore, AFM technology was also selected to systematically research the microscopic properties of UV-aged asphalt in this paper. The changing characteristics of the asphalt’s microscopic structure during the aging process will be meticulously analyzed, encompassing the evolution laws of the area, elevation, and surface roughness of the bee structure. Moreover, an attempt will be made to establish a mathematical model between the microscopic structure parameters and the macroscopic performance indicators of asphalt binder, leveraging the LM and UGO algorithms within the realm of nonlinear algorithms. It is anticipated that this endeavor will afford a profound understanding of the influence mechanism of UV aging on the microscopic structure and properties of both SBS-modified asphalt and base asphalt.

It should be clearly stated that the paper is primarily focused on the nanoscale properties of the asphalt binders before and after UV aging, which could be adequately and clearly characterized in quantity by atomic force microscopy (AFM). High accuracy and good repeatability could be guaranteed in measured data by the test and shows unique advantages in quantitatively characterizing the mechanical parameters and UV aging process of asphalt binder compared with other test methods, such as SEM (scanning electron microscope) [24,34,35,36]. Therefore, based on the considerations discussed above, AFM was employed in this paper.

## 2. Materials and Methods

### 2.1. Material

The materials selected in this paper are Donghai brand 70# base asphalt and SBS (I-D)-modified asphalt. The technical indicators of the two asphalts are shown in Table 1 according to (JTG E20-2011) [37].

Various indicators of base asphalt and SBS-modified asphalt were tested, as illustrated in Table 1. All indicators meet the requirements of the specification.

### 2.2. Test Method

**Figure 1:**
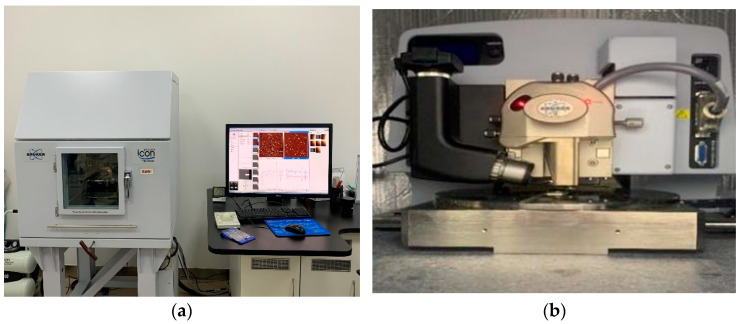
The process of the AFM test. (**a**) AFM apparatus. (**b**) AFM testing.

Atomic force microscopy (AFM) was selected to test the morphology of base asphalt and SBS-modified asphalt after different UV aging times. The test process is shown in Figure 1. The AFM morphology test selected the SCANASMST-AIR type probe, and the probe parameters are shown in Table 2.

AFM samples were prepared using the hot-melt method. The preparation process was as follows: First, a metal ring with an outer diameter of 17 mm, an inner diameter of 15 mm, and a height of 1 mm was placed on the glass sheet. Then, the heated asphalt was poured into the ring to make the asphalt slightly higher than the metal ring to ensure the smooth surface of the asphalt. Finally, it was cooled to room temperature in a sealed sample dish.

Short-term aging was first carried out by rotating thin film oven aging test (RTFOT), and then the two kinds of asphalt were aged for 2 months (2 M), 4 months (4 M), 8 months (8 M), and 12 months (12 M) after RTFOT. This test protocol was designed to evaluate the performance of base and SBS-modified bitumen during long-term UV aging.

## 3. Results and Discussion

**Figure 2:**
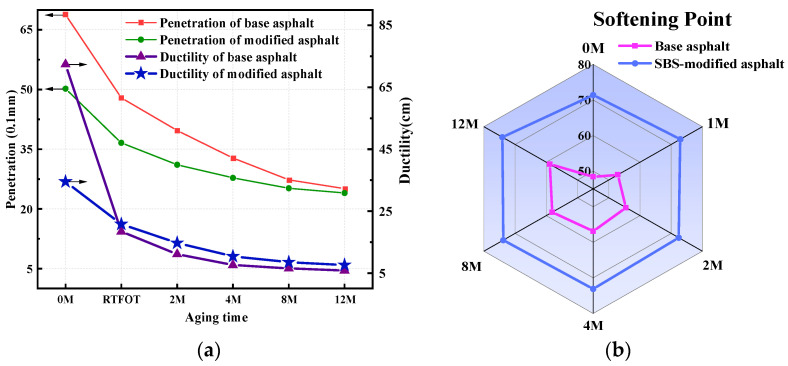
Performance index of aged asphalt. (**a**) Asphalt penetration. (**b**) Asphalt softening point.

The technical indexes of asphalt after different ultraviolet aging times are shown in Figure 2.

It can be seen from Figure 2 that as the aging time extends, the penetration and ductility of the two kinds of asphalt gradually diminish, while the softening point gradually rises. Following the RTFOT, the penetration and ductility of base asphalt decreased significantly. After 12 M UV aging, the performance indexes of asphalt become identical, basically. In comparison with the base asphalt, with the UV aging time increasing, the changing trend of the softening point of SBS-modified asphalt is not significant, which proves the advantage of the anti-UV aging performance of SBS-modified asphalt.

### 3.1. Morphology and Microstructure

**Figure 3:**
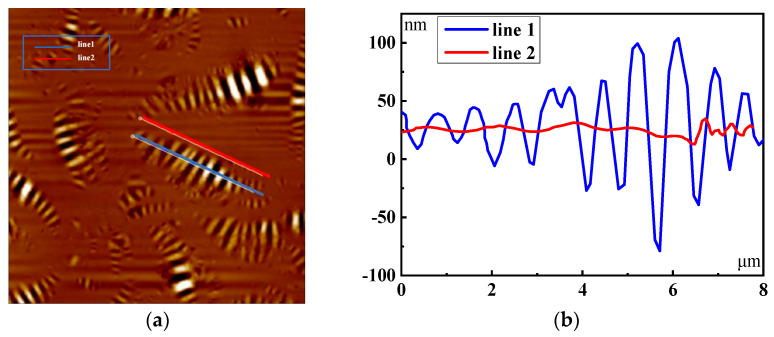
Asphalt surface height profile. (**a**) Selection area. (**b**) Two-dimensional elevation.

The morphology and structure of asphalt will change with the increase in UV aging time, and the bee structure will directly affect the absolute elevation value of the asphalt surface. Therefore, the AFM intelligent scanning mode is used to obtain the microscopic data of the asphalt mixture under different aging times. The width module is used to extract the absolute elevation value of the bee structure and its surrounding area. The selected area is shown in Figure 3a, and the two-dimensional elevation maps at different positions are shown in Figure 3b.

It can be seen in Figure 3a that the bee structure is distributed throughout the whole structure. Due to the influence of the components, the elevation of the bee structure fluctuates significantly, while the elevation of the non-bee structure is basically the same and the region is smooth. Therefore, the existence of the bee structure is an important factor affecting the microscopic behavior of asphalt.

**Figure 4:**
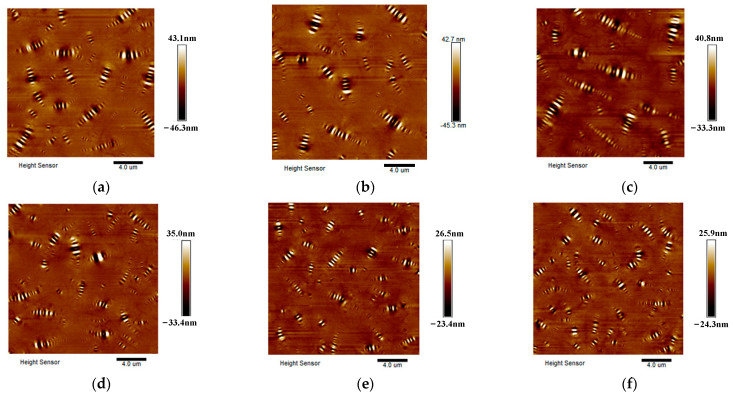
Elevation map of base asphalt under different UV aging times. (**a**) 0 M, (**b**) RTFOT, (**c**) 2 M, (**d**) 4 M, (**e**) 8 M, (**f**) 12 M.

**Figure 5:**
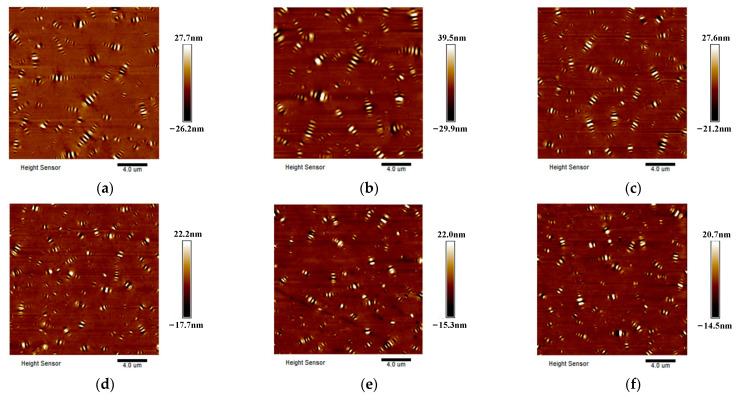
Elevation map of SBS-modified asphalt under different UV aging times. (**a**) 0 M, (**b**) RTFOT, (**c**) 2 M, (**d**) 4 M, (**e**) 8 M, (**f**) 12 M.

In order to analyze the anti-UV aging performance of the two kinds of asphalt from the bee structure, the morphology of the two kinds of asphalt under different UV aging times was selected and analyzed, as shown in Figure 4 and Figure 5.

In comparison with Figure 4 and Figure 5, it was found that the number of bee structures of SBS-modified asphalt is significantly more than that of base asphalt in the whole process of UV aging. Following the RTFOT, in comparison with the state before aging, the number of bee structures in the two types of asphalts did not exhibit a significant alteration. However, with continuous UV aging, the larger bee structure gradually disappeared, and the bee structure gradually became smaller and compact.

**Figure 6:**
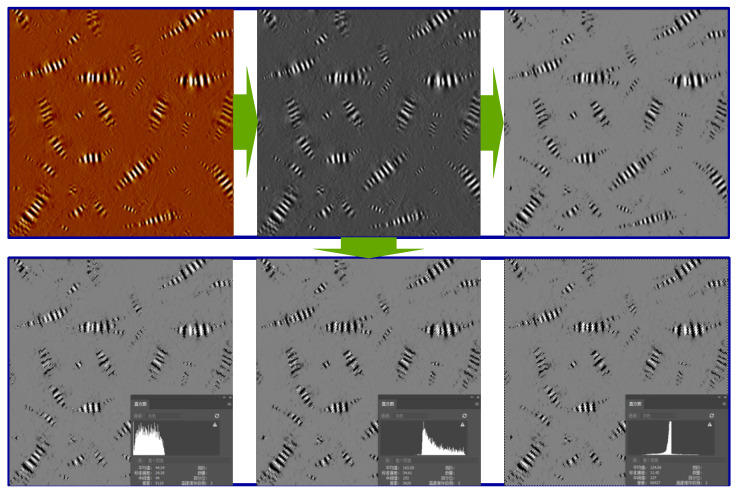
Gray image process.

To further analyze the effect of UV aging on the performance of asphalt from the perspective of the bee structure, it was gray-scale processed, and the proportion of black and white colors in the image was calculated. The image process is shown in Figure 6.

Figure 6 initiates with a color transformation process on the image, thereafter proceeds to feature extraction, and subsequently computes and analyzes the spatial distribution and number of black, white, and gray hues within the image. The area and number of two kinds of asphalt bee structures under different UV aging times were calculated as shown in Table 3.

It can be seen from the data in Table 3 and Figure 6 that with the increase in UV aging time, the white area representing asphaltene and the black area representing saturate gradually decrease, while the gray area gradually increases. The bee structure area of the two kinds of asphalt is becoming smaller and smaller with the increase in ultraviolet aging time. The number of bee structures of the base asphalt is much less than that of the SBS-modified asphalt, and the area proportion of the bee structure of the two kinds of asphalt is basically the same. It is inferred that the number of bee structures will directly affect the anti-UV aging performance of asphalt. The more bee structures there are, the better the anti-aging performance of asphalt.

### 3.2. Roughness Feature Quantification

Through the AFM test, it is found that the surface of the asphalt was uneven, which was completely different from the macroscopic phenomenon. The results show that the change in surface structure of asphalt under UV light irradiation can directly reflect the change in asphalt roughness during aging, and the roughness of asphalt surface is closely related to its bonding performance.

**Figure 7:**
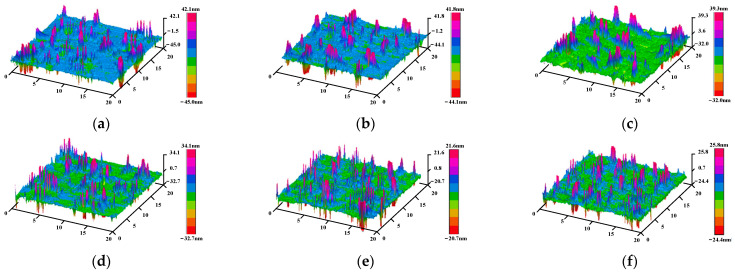
Three-dimensional elevation map of base asphalt at different aging times. (**a**) 0 M, (**b**) RTFOT, (**c**) 2 M, (**d**) 4 M, (**e**) 8 M, (**f**) 12 M.

**Figure 8:**
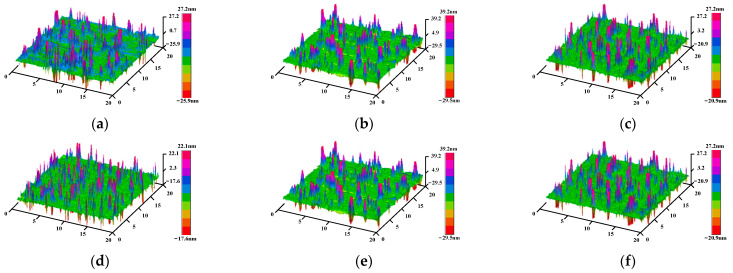
Three-dimensional elevation map of SBS-modified asphalt at different aging times. (**a**) 0 M, (**b**) RTFOT, (**c**) 2 M, (**d**) 4 M, (**e**) 8 M, (**f**) 12 M.

Rq (height root mean square) and Ra (arithmetic mean deviation of contour) were selected as the indexes to evaluate the roughness of asphalt, so as to characterize the evolution process of the asphalt microstructure under different UV aging times. The three-dimensional elevation diagram of the two kinds of asphalt under different ultraviolet aging time is shown in Figure 7 and Figure 8.

As can be observed from Figure 7 and Figure 8, the flatness of base asphalt and SBS-modified asphalt remains relatively good before and after aging. Following RTFOT, the distance between the peak and the bottom of the SBS-modified asphalt increases, whereas that of the base asphalt decreases. With the deepening of UV aging, the peak value changes slightly, but the difference between the peak and valley of the two asphalts is shrinking. Through comparison, it is noted that the difference between the peak and valley of the base asphalt during the UV aging process is greater than that of the SBS-modified asphalt, which shows that the base asphalt is more susceptible to UV aging, and the SBS-modified asphalt has better UV aging resistance. The calculated roughness parameters of the two kinds of asphalt after different ultraviolet aging durations are presented in Table 4.

It can be seen from Table 4 that after RTFOT and UV aging, the roughness of the base asphalt decreases continuously. The roughness of the SBS-modified asphalt before and after aging is less than 3 nm, and the surface is flat. After RTFOT, the roughness increases by 40%. However, in the early stage of UV aging, the roughness decreases greatly. With the increase in UV aging time, the roughness of the SBS-modified asphalt also decreases continuously. UV radiation will directly affect the proportion of each component of asphalt. The light components in asphalt will continuously transform into resins under the action of UV aging, while resins will continue to change to asphaltenes, resulting in an increase in the proportion of asphaltenes. The difference between the components of the asphalt gradually decreases, so that the roughness of the asphalt after aging continues to decrease [38].

Combined with the analysis of the change process of the 3D elevation map during the UV aging process, the asphaltene content increases during the short-term aging process and overlaps with the SBS modifier molecule in the physical structure, resulting in a larger peak-to-valley spacing of the bee structure, a larger elevation gap with the smooth area, and an increase in surface roughness. In the early stage of UV aging, the SBS modifier absorbs UV energy and degrades, resulting in a decrease in the elevation of the larger bee structure, and the overall homogenization, so the roughness is small. With the increase in UV aging time, the content of asphaltene is increasing, and the surface fluctuation is less obvious, so the roughness is decreasing.

**Figure 9:**
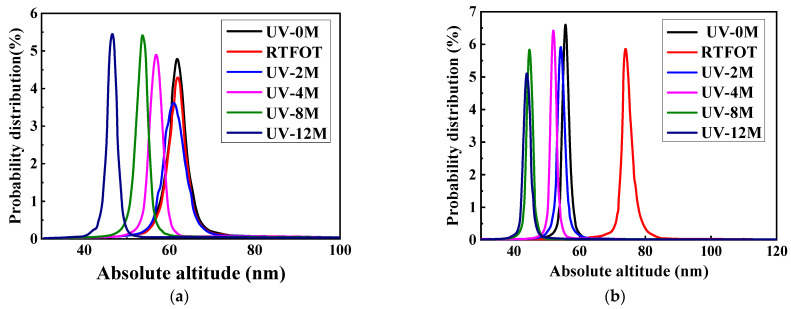
Maps of the absolute height distribution of the two kinds of asphalt. (**a**) Base asphalt, (**b**) SBS-modified asphalt.

In order to further analyze the changing trend of asphalt micro-surface elevation, the data on asphalt micro-surface height are extracted by the width module, and the height frequency distribution map is drawn. As shown in Figure 9, the distribution rate of two kinds of asphalt surface height under different ultraviolet aging times is shown in the figure.

As shown in Figure 9, after the base asphalt undergoes RTFOT, the absolute height of the bee structure remains nearly unchanged, but its number decreases. During the continuous UV aging process, the absolute height of the bee structure steadily decreases while its distribution frequency continuously rises. This suggests that after UV aging, the surface of the base asphalt is smoother, and the number of bee structures is increasing. Although the SBS-modified asphalt exhibits a similar trend in the bee structure as the base asphalt, its height distribution range is narrower than that of the base asphalt, implying a more consistent height of the bee structure in the SBS-modified asphalt. After RTFOT, the absolute height of the bee structure of the SBS-modified asphalt increased significantly. However, after UV aging 2 M, it drops significantly and becomes basically identical to that of the unaged asphalt. As the UV aging time extends, the absolute height of the bee structure keeps declining.

### 3.3. Adhesion Force Characteristics

**Figure 10:**
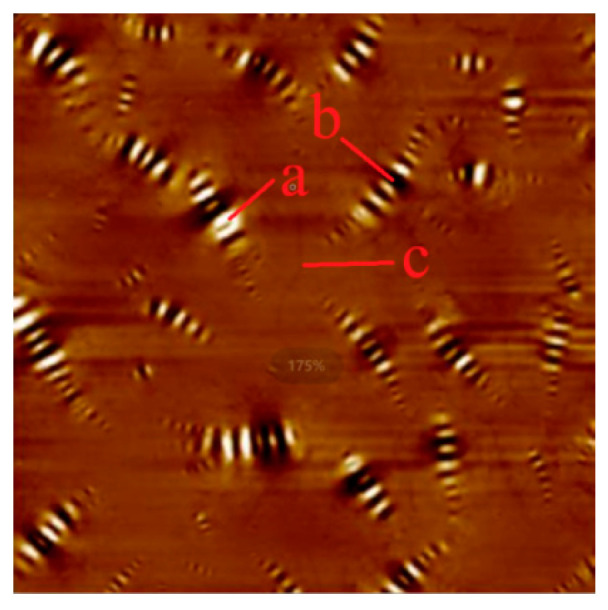
Asphalt adhesion test point selection schematic diagram. (a) white area, (b) black area, (c) smooth area.

In order to analyze the change process of asphalt adhesion, the PF-QNM force measurement mode was used to test nine force curves on the white area (a), black area (b), and smooth area (c) of the asphalt bee structure, as shown in Figure 10. Then, six similar force curves are selected, respectively. Considering the area of different parts of the bee structure and the area of the smooth area in the asphalt topography, the overall adhesion of the asphalt is calculated, as shown in Equation (1).

(1) $$F_{ad-whole}=S_{a}\times F_{a}+S_{b}\times F_{b}+S_{c}\times F_{c}$$

where *F_ad-whole_* represents the overall adhesion of asphalt, nN. S_i_ represents the proportion of area in different regions, %, i = a, b, c. F_i_ represents the adhesion force of different regions, nN, i = a, b, c.

The adhesion data of the base asphalt and SBS-modified asphalt in different regions and the overall adhesion data of the asphalt after different aging time are shown in Table 5.

As shown in Table 5, through weighted calculation, it can be observed that the overall adhesion force of the base asphalt and SBS-modified asphalt has a good regularity with aging time. The adhesion force of the smooth region is slightly higher than that of the bee structure, and the adhesion force of the peak and valley has no significant regularity. In general, the overall adhesion of the two kinds of asphalt gradually decreases. The adhesion of the SBS-modified asphalt decreases rapidly after RTFOT, but the adhesion in the early stage of UV aging is the same as that in the RTFOT. With the increase in UV aging time, the overall adhesion gradually decreases. Therefore, under the influence of UV aging, the aging speed of SBS-modified asphalt shows the law of rapid aging, slow aging, and rapid aging. Due to the significant impact of aging on the base asphalt, its adhesion force exhibits a downward tendency through the entire aging process.

**Figure 11:**
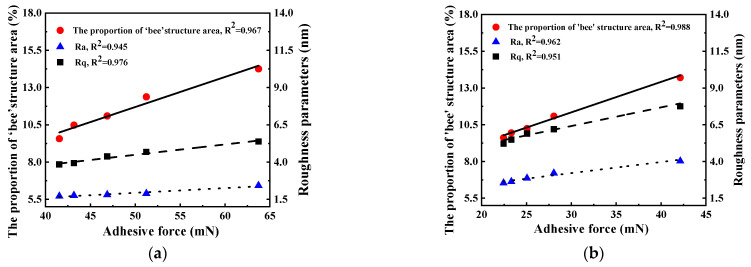
Linear analysis of adhesion force and bee structure area and roughness. (**a**) Base asphalt, (**b**) SBS-modified asphalt.

The relationship among the bee structure area, roughness, and adhesion of asphalt during different aging processes is shown in Figure 11.

As depicted in Figure 11, a distinct linear correlation exists between the variation in the adhesion force of the two kinds of asphalt and the roughness parameters, Ra and Rq, as well as the area of the bee structure. This indicates that under the influence of UV aging, the micro-adhesion force of asphalt is intrinsically linked to the reduction in the area of the bee structure and the changes in the roughness parameters of the asphalt.

As the bonding force and roughness of asphalt diminish, the surface of asphalt will grow flatter and display a stronger tendency towards being ‘uniform’. When the asphalt is aged by UV light, the resin content within the asphalt progressively transforms into asphaltene. This conversion leads to a substantial reduction in the effective contact area of the asphalt, subsequently causing a decline in its adhesion force. The linear correlation between the adhesion force and the adhesion force is presented in Figure 11. Therefore, it can be inferred that the change in asphaltene content triggers the alteration of the bee structure area in asphalt.

Combined with the above analysis, it is concluded that the aromatic components in asphalt will undergo oxidative polymerization to form colloids under the action of UV aging, and the polar functional groups in the colloid will undergo condensation reaction during UV aging and gradually transform into asphaltenes. With the increase in asphaltenes, the adhesion of asphalt decreases; due to the presence of SBS modifier molecules, SBS-modified asphalt reduces the contact between asphalt molecules and UV rays and delays the aging process of asphalt. Therefore, SBS-modified asphalt has better adhesion under the same aging conditions.

**Figure 12:**
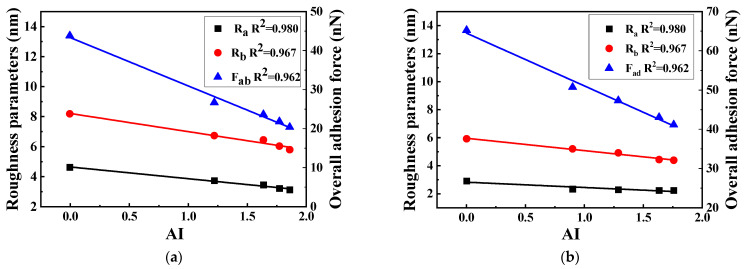
The correlation diagram of AI and AFM factors. (**a**) Base asphalt, (**b**) SBS-modified asphalt.

**Figure 13:**
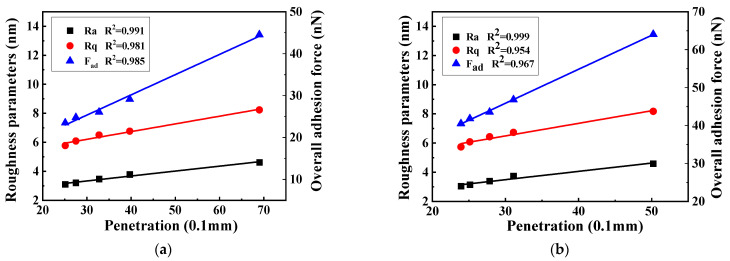
Penetration and AFM factor correlation diagram. (**a**) Base asphalt, (**b**) SBS-modified asphalt.

The above studies have concluded that the macroscopic properties of base asphalt and SBS-modified asphalt have changed after UV aging. In order to explore the relationship between the basic indicators of asphalt and the AFM test results, penetration, aging index (AI), roughness, and adhesion were selected for analysis. The calculation formula of AI is shown in Equation (2) [14], and the change process of asphalt performance index is shown in Figure 12 and Figure 13.

(2) $$AI=\frac{\left|G^{*}\right|/\sin\delta_{afterUVaged}}{\left|G^{*}\right|/\sin\delta_{unaged}}$$

where $\left|G^{*}\right|/\sin\delta_{afterUVaged}$ and $\left|G^{*}\right|/\sin\delta_{unaged}$ are the parameter G*/sin*δ* of the aged and unaged asphalt, respectively. G*/sin*δ* is the rutting factor. G* is the complex shear modulus. *δ* is the phase angle.

From Figure 12 and Figure 13, it can be observed that the penetration and AI of the two kinds of asphalt under different ultraviolet aging times have an obvious linear relationship with AFM microscopic index, and R^2^ is above 0.84. This shows that the macroscopic physical properties and rheological properties of asphalt under different UV aging times are closely related to the microstructure and nanomechanics.

### 3.4. Nonlinear Prediction of UV Aging

Studies have indicated that asphalt aging exhibits saturated state characteristics. Specifically, at the commencement of UV aging, the aging rate of asphalt is at its peak. As the aging time extends, asphalt molecules keep polymerizing, and the number of asphalt molecules capable of reacting with oxygen gradually decreases, which causes the aging process of asphalt to decelerate until it attains a saturated state, at which point the aging process halts. The Verhulst model [39] can be used to describe the aging process with the saturated state characteristics, as presented in Equation (3).

(3) $$x^{\prime }(t)=\alpha x(t)-\beta x^{2}(t)$$

where *x*(*t*) is the asphalt performance at t time. x′(t) is the change rate of asphalt performance at t time. *A* and *β* are constants.

By separating variables, the solution for Equation (3) is as follows:

(4) $$x(t)=\frac{\alpha }{\beta (1-Ce^{-\alpha t})}$$

where *C* is a constant. When *t* = 0, *x*(*t*) = *x*_0_, then *C* = 1 − *α*/(*βx*_0_). Let *α/β = K*, then, the following occurs:

(5) $$x(t)=\frac{k}{1+(\frac{k}{x_{0}}-1)e^{-\alpha t}}$$

Let $\frac{k}{x_{0}}=L$, α=r then, the following occurs:

(6) $$x(t)=\frac{Lx_{0}}{1+(L-1)e^{-rt}}$$

where when *t*→∞, *x*(*t*)→*Lx*_0_, namely, *L* = lim*t*→∞*x*(*t*)/*x*_0_. The parameter *L* is the ratio of the final performance and the initial performance of the aged asphalt, and its value represents the final aging state of the asphalt; r is the aging rate of the asphalt.

In order to establish the relationship between the aging time and the performance of asphalt materials, the damping Newton algorithm and the general global optimization algorithm are selected to analyze the relationship between the data. The UGO algorithm does not need to input the initial value when fitting, which solves the problem that the initial value cannot be determined in the actual calculation process. The LM algorithm avoids the limitation that the parameter distribution generally obeys the normal distribution when estimating the parameters.

Taking LM algorithm as an example, the calculation process of the UV aging nonlinear prediction equation is described. Firstly, we assume that the function *y = f*(*t*;*θ*) can characterize the relationship between the asphalt performance index and aging time, where *θ* is the parameter vector to be estimated; the initial value of the adhesion force of asphalt without full climate aging is assumed to be y_0_ = f(0;θ). The asphalt performance after UV aging 12 M is set as the final value y_12_ = f(12;θ), parameter $L=\frac{y_{12}}{y_{0}}$; the objective function can be written as follows:

(7) $$J(\theta )={\sum_{i}\left[y_{i}-f(t_{i};\theta )\right]}^{2}$$

where y_i_ is the measured asphalt performance, and *t_i_* is the aging time.

The iterative formula can be expressed as follows:

(8) $$\theta_{n+1}=\theta_{n}-H^{-1}(\theta_{n})\nabla J(\theta_{n})$$

In the formula, *θ_n_* is the parameter vector of the nth iteration, $\nabla J(\theta_{n})$, which is the gradient vector of the objective function *J*(*θ*) at *θ_n_*, and H(*θ_n_*) is the Hessian base of the objective function *J*(*θ*) at *θ_n_*.

Calculate the gradient according to Equation (9).

(9) $$\nabla J(\theta_{n})=\sum_{i}-2[y_{i}-f(t_{i};\theta )]\frac{\partial f(t_{i};\theta )}{\partial \theta }$$

Calculate the Hessian base according to Equation (10).

(10) $$H(\theta )=\sum_{i}2\times \frac{\partial f(t_{i};\theta )}{\partial \theta }\times (\frac{\partial f(t_{i};\theta )}{\partial \theta }{)}^{T}-2[y_{i}-f(t_{i};\theta )]\frac{\partial^{2}f(t_{i};\theta )}{\partial \theta \partial \theta^{T}}$$

Assuming that the damping factor is *λ*, the iterative formula becomes the following:

(11) $$\theta_{n+1}=\theta_{n}-\lambda H^{-1}(\theta_{n})\nabla J(\theta_{n})$$

Through continuous iteration until the convergence condition is satisfied, the relationship function among the asphalt performance index and aging time and related parameters are determined. The fitting results are shown in Table 6.

As can be observed from the aging equation of each AFM index in Table 6, the r value of SBS-modified asphalt is considerably smaller than that of base asphalt. This implies that the addition of the modifier remarkably enhances the anti-aging ability of the asphalt, with the SBS-modified asphalt exhibiting a relatively low aging rate.

With the extension of UV aging time, the micro-morphology values of both the SBS-modified and base asphalt decrease, while their adhesion and modulus values increase gradually. The correlation coefficients of roughness and adhesion of the two asphalts are 0.979 and 0.985 (morphology height), and 0.951 and 0.934 (for adhesion), respectively. Hence, this equation can be utilized to characterize the alterations in the micro-morphology and micro-performance of asphalt aging under UV aging.

## 4. Conclusions

In this study, atomic force microscopy was utilized to perform a profound and comprehensive analysis of the alterations in performance indices of SBS-modified asphalt and base asphalt under different UV aging times. Subsequently, the inherent mechanism responsible for the degradation of asphalt performance induced by UV aging was effectively disclosed. At the same time, based on the powerful functions of the LM algorithm and the UGO algorithm, an effective approach for accurately evaluating the micro-morphology and micro-performance changes in asphalt under UV aging conditions was constructed. The main conclusions are as follows:

(1) The bee structure is closely related to asphalt performance. Throughout the entire aging process, the quantity of bee structures in SBS-modified asphalt is substantially greater than that in base asphalt. UV aging affects the performance of asphalt by changing the number and area of the bee structure in the asphalt. As UV aging progresses, the area of bee structure in asphalt gradually decreases, and the distance between the peak and the valley gradually decreases.
(2) The specific performance is that the roughness of SBS-modified asphalt is relatively stable after short-term aging, and the absolute height of the bee structure experiences a slight increment. Nevertheless, as the UV aging time lengthens, the disparity between the peak and valley of the bee structure of both types of asphalt diminishes. The absolute height of the bee structure keeps dropping, and the surface roughness of the asphalt is gradually decreasing. Notably, the height distribution range of the bee structure in SBS-modified asphalt is narrower and more uniform.
(3) The overall adhesion of asphalt exhibits clear regularity in the relation to the aging time, and the overall adhesion of the two kinds of asphalt decreases with the increase in aging time. As is different from the change rule of base asphalt adhesion, the adhesion of SBS-modified asphalt increases slightly from short-term aging to aging 2 M. Subsequently, with the growth of UV aging time, the adhesion of asphalt decreases progressively.
(4) For the two kinds of asphalt, the penetration and AI under different UV aging durations display an evident linear relationship with the AFM microscopic index. Based on the LM and UGO algorithms, the calculation equations of the morphology height and adhesion force of SBS-modified asphalt and base asphalt under different aging time are obtained. These equations can proficiently characterize the alterations in the micro-morphology and micro-performance of asphalt under UV aging conditions.

## Figures and Tables

**Table 1:** Technical index of Donghai brand 70 # base asphalt and SBS (I-D)-modified asphalt.

Performance Index		Quality Index	Test Result	Quality Index	Test Result	Testing Standards
		Base Asphalt		SBS-Modified Asphalt		
Penetration (25 °C, 100 g, 5 s) (0.1 mm)		60~80	71.6	40~60	51.6	T0604
Ductility (5 cm/min, 15 °C) (cm)		≥100	>100	≥20	32	T0605
Soft pointing (ball and ring method) (°C)		≥46	47.3	≥60	75.0	T0606
Density (15 °C) (g/cm^3^)		measurement	1.036	measurement	1.024	T0603
Solubility (trichloroethylene) (%)		≥99.5	99.80	≥99	99.90	T0607
Storage stability segregation, 48 h softening point difference (°C)		-	-	≤2.5	1.6	T0661
RTFOT 163 °C, 85 min	Quality loss (%)	≤±0.8	0.08	≤±1.0	0.222	T0610
	Residual penetration ratio (25 °C) (%)	≥61	63.6	≥65	72	
	Ductility (5 cm/min, 15 °C) (cm)	≥6	7	≥15	18	

**Table 2:** SCANASMST-AIR probe parameters.

Probe Model	Tip Radius/nm	Frequency/kHz	Stiffness Factor N/m	Cantilever Number	Back Coating
SCANASMST-AIR	2	70	0.4	1	Reflective aluminum

**Table 3:** The change in area and number of the bee structure of the two asphalts with different UV aging times.

Aging Time	Area (%)			Area	Number
	White	Black	Gray		
Base asphalt					
0 M	6.41	7.30	86.29	13.71	29
RTFOT	5.92	6.09	87.99	12.01	31
2 M	5.84	5.25	88.91	11.09	32
4 M	5.34	4.91	89.75	10.25	42
8 M	5.32	4.63	90.05	9.95	48
12 M	5.06	4.56	90.38	9.62	56
SBS-modified asphalt					
0 M	5.78	6.48	87.74	12.26	65
RTFOT	7.75	6.83	85.42	14.58	66
2 M	6.05	6.33	87.62	12.38	76
4 M	5.23	5.91	88.85	11.14	89
8 M	5.32	5.66	89.02	10.98	84
12 M	4.94	4.63	90.43	9.57	89

**Table 4:** Roughness changes in two kinds of asphalt after different ultraviolet aging times.

Aging Time	Base Asphalt				SBS-Modified Asphalt			
	Ra	SD	Rq	SD	Ra	SD	Rq	SD
0 M	4.03	0.21	7.66	0.68	2.42	0.20	5.38	0.36
RTFOT	3.48	0.19	7.03	0.38	3.39	0.35	7.81	2.21
2 M	3.21	0.22	6.20	0.20	1.83	0.10	4.68	0.17
4 M	2.86	0.40	5.90	0.96	1.81	0.08	4.38	0.16
8 M	2.63	0.14	5.49	0.26	1.76	0.13	3.93	0.36
12 M	2.54	0.07	5.22	0.01	1.71	0.19	3.84	0.27

**Table 5:** The change in overall adhesion of the base asphalt and SBS-modified asphalt at different aging times.

Aging Time	Proportion of Area in Different Regions/%			Adhesion in Different Regions Fad/nN			Overall Adhesion Force Fad/nN
	Peak Maximum	Peak Trough	Smooth Region	Peak Maximum	Peak Trough	Smooth Region	
Base asphalt							
UV-0 M	6.41	7.30	86.29	41.19	39.68	43.18	42.79
RTFOT	5.92	6.09	87.99	30.78	31.25	32.15	32.01
UV-2 M	5.84	5.25	88.91	27.43	26.1	28.21	28.05
UV-4 M	5.34	4.91	89.75	24.74	24.66	25.34	25.27
UV-8 M	5.32	4.63	90.05	22.61	22.67	23.98	23.85
UV-12 M	5.06	4.56	90.38	20.90	20.08	22.97	22.73
SBS-modified asphalt							
UV-0 M	5.78	6.48	87.82	62.95	61.57	63.88	63.73
RTFOT	7.75	6.83	85.42	46.57	47.41	49.77	50.29
UV-2 M	6.05	6.33	87.62	47.63	49.78	51.24	50.43
UV-4 M	5.23	5.91	88.91	43.33	41.78	47.63	46.87
UV-8 M	5.32	5.66	89.02	39.1	41.8	43.49	43.17
UV-12 M	4.94	4.63	90.43	39.57	39.20	41.77	41.54

**Table 6:** Two kinds of asphalt parameters and their aging equations.

The Type of Asphalt	AFM Index	r (10^−5^)	L	Aging Equation	R^2^
Base asphalt	Roughness	52.787	0.283	H = 1.37/(1 − 0.72 × 10^−0.00052782^ t)	0.979
	Adhesion	118.763	3.974	A = 0.87/(1 + 2.97 × 10^−0.00118763^ t)	0.951
SBS-modified asphalt	Roughness	9.3291	0.068	H = 0.36/(1 − 0.93 × 10^−0.000093291^ t)	0.985
	Adhesion	82.126	3.793	A = 0.98/(1 + 2.79 × 10^−0.00082126^ t)	0.934

## Data Availability

Data can be provided upon request.
